# Optimization of Rheological Behaviour and Skin Penetration of Thermogelling Emulsions with Enhanced Substantivity for Potential Application in Treatment of Chronic Skin Diseases

**DOI:** 10.3390/pharmaceutics11080361

**Published:** 2019-07-24

**Authors:** Markus Schmidberger, Ines Nikolic, Ivana Pantelic, Dominique Lunter

**Affiliations:** 1 Department of Pharmaceutical Technology, Eberhard Karls University, Auf der Morgenstelle 8, 72076 Tuebingen, Germany; 2 Department of Pharmaceutical Technology and Cosmetology, Faculty of Pharmacy, University of Belgrade, 450 Vojvode Stepe Street, 11221 Belgrade, Serbia

**Keywords:** nonivamide, methyl cellulose, skin penetration, substantivity, thermogel

## Abstract

Topical formulations are an important pillar in the therapy of skin diseases. Nevertheless, after application the formulation will be exposed to environmental effects. Contact with other surfaces will reduce the available amount of formulation and drug substance. The resulting consequences for therapy range from reduced effects to therapeutic failure. The removed active ingredient also contaminates patients’ environment. The aim of this work was to develop preparations that remain at the application site. These will enhance safety and efficiency and thus improve of skin disease therapies. Therefore, we developed polymer-stabilised emulsions that show thermogelling properties. Emulsions with different methyl cellulose concentrations and macrogols of different molecular weights were investigated. The dispersed phase consisted of nonivamide as the active pharmaceutical ingredient, dissolved in medium-chain triglycerides. Rheological properties, droplet size, substantivity and ex vivo penetration experiments were performed to characterise the developed formulations. Droplet size and rheological parameters were affected by the composition of the preparations. The tested formulations showed benefits in their substantivity compared to a conventional semi-solid cream. We found a residual amount of up to 100% at the application site. The drug levels in viable epidermis were in a therapeutic range. The developed emulsions are a promising vehicle to improve therapy for chronic skin diseases.

## 1. Introduction

The vehicle of a topical formulation plays an important role with respect to disease control and therapeutic outcome. It controls the penetration profile of the preparation [1,2,3,4,5] and has an effect on the skin barrier function [6,7]. This can improve or worsen the healing process of diseased skin. However, the applied formulation is partly removed, e.g., by touching skin surfaces or clothing. As a result, the drug and vehicle can be removed from the application site and thus cannot achieve the desired outcome. Higher drug concentration or repeated application will conceal this problem but will not solve it. Additionally, active pharmaceutical ingredients (API) can pollute the patients’ environment. Consequently, other people can come into contact with the drug substance, resulting in irritations or undesired effects. Topical formulations that are more resistant to contact with the environment and remain at the application site are a promising approach to improve patients’ compliance and reduce the consumption of such formulations. Safety aspects are another benefit. If the preparation stays at the application site, unintentional contact will be minimised. Furthermore, formulations that remain on the skin surface are possible drug reservoirs for topical preparations that exhibit sustained release.

The ability of a topical formulation to remain on the skin surface is called substantivity [8]. It has been shown that different types of formulations show notable differences in their substantivity. A substantial amount of semi-solid formulations and liquid preparations are removed from the skin surface by contact with other surfaces, whereas film-forming formulations are not affected [9,10]. These preparations differ in their rheological properties. Thus, it is a promising approach to tune the rheological behaviour of a topical formulation to increase its substantivity. We hypothesise that formulations that change their rheological behaviour to a more elastic character upon application to the skin, e.g., by thermogelation, will show enhanced substantivity. We thus developed emulsions that are stabilised with methyl cellulose (MC). Cellulose derivates, such as hydroxypropyl methylcellulose or methyl cellulose, are known to be suitable for preparing oil-in-water emulsions and show thermogelation [11,12,13,14,15].

In this work, we focus on nonivamide as a drug substance for chronic skin diseases. The compound is an agonist on the transient receptor vanilloid type 1 ion channel (TRPV1) [16]. This non-specific ion channel is present in human skin, especially in sensory nerve fibres [17]. The short-term activation of TRPV1 results in different symptoms. Most pronounced are warming and burning, in addition to stinging and itching [18,19]. With long-term use, the intracellular amount of calcium increases due to calcium influx and after a certain time the cellular processes modify. As a result, neurons are defunctionalised and the stimulus of itch is not conducted. Therefore, nonivamide and other capsaicinoids have a certain analgesic and antipruritic potential. They are thus agents used to treat neuralgic pain or chronic pruritus [20,21], which presents in many skin diseases. Their use in clinical practise is limited due to the necessity of multiple dosing and thus impaired patient compliance. Formulations with enhanced substantivity might therefore offer a beneficial approach to promote the use of capsaicinoids.

The substantivity of dermal preparations is less investigated and little is known about the influencing factors. A further aim of this work was thus to understand the rheological factors that influence substantivity and to employ this knowledge in the development of a topical formulation that remains at the application site due to optimised rheological properties.

## 2. Material and Methods

### 2.1. Materials

Methyl cellulose (Metolose SM-25) was kindly donated by Shin-Etsu Chemical Co., Tokyo, Japan. Avobenzone was provided by Symrise AG, Holzminden, Germany. All other materials were obtained from the named supplier: sodium citrate (Carl Roth GmbH + Co. KG, Karlsruhe, Germany), nonivamide (Sigma Aldrich Chemie GmbH, Steinheim, Germany), white soft paraffin (Hansen and Rosenthal KG, Hamburg, Germany), medium-chain triglycerides (Myritol^®^ 312), propylene glycole and macrogol 200 and 4000 (BASF SE, Ludwigshafen, Germany) and cetyl alcohol (Ceasar and Loretz, Hilden, Germany). PEG-20-glyceryl stearate (Tagat S2^®^) and glycerol stearate (Fagron GmbH and Co. KG, Barsbüttel, Germany). Solvents methanol and acetonitril were HPLC gradient grade. Sodium chloride, disodium phosphate, potassium dihydrogen phosphate, magnesium sulphate and phosphoric acid were of European Pharmacopeia grade.

### 2.2. Preparation of Oil-in-Water Emulsions

The formulation was prepared by a syringe-to-syringe technique. Methyl cellulose, long-chain macrogol and sodium citrate were weighed into a syringe. Water was heated up to boiling and then weighed into a second syringe of the same size. The water had a temperature of 85–90 °C. Water-miscible liquids such as ethanol or liquid (short-chain) macrogol were added to the hot water. The two syringes were connected by an adapter and then mixed until no solid particles were visible. A white dispersion was the result. To avoid the incorporation of air in the formulation, the syringes were disconnected and the air in the headspace was removed. Subsequently, the syringes were connected again and the dispersion was mixed 100 times by transferring the mixture from one syringe to the other. The formulation was stored at 5 °C for 24 h. The preparation of such aqueous methyl cellulose preparations has already been described by Takeuchi et al. [22]. As a final step, nonivamide was dissolved in medium-chain triglycerides. The oily solution was weighed into a syringe and connected to the prepared aqueous phase. For a preparation with 0.9% API, the concentration of the oily solution was 3.6% nonivamide in medium-chain triglycerides. For an emulsion with 0.3% nonivamide, the concentration of API in the lipophilic phase was 1.2%. The two phases were mixed 100 times. As a result, a white oil-in-water emulsion was formed. The percentage composition of the emulsions is given in Table 1. The liquid formulation was filled into a glass vial and tightly sealed. Due to the preparation in a closed system, it was not necessary to add evaporated water.

Formulations used in the in vivo substantivity measurements contained avobenzone instead of nonivamide. Formulations were prepared as described above.

### 2.3. Rheoloigcal Measurements

A rotational viscometer (Physica MCR 501, Anton Paar, Graz, Austria) with a plate/plate measuring arrangement (*d* = 25 mm) was used to characterise the rheological behaviour of the formulations. Oscillatory tests were performed at 5 °C and 32 °C. A temperature of 5 °C ensured that the measurement was below the gelling point. The rheological behaviour on skin surface was determined at 32 °C. The frequency was 1 Hz and the deformation was increased from 0.01 to 1000%. The gap size was set to 0.2 mm. Storage modulus G’ and loss modulus G’’ were determined in the linear viscoelastic region. The dissipation factor was calculated as shown in Equation (1). All formulations contained 0.9% nonivamide. Experiments were performed in triplicate.

(1) $$Dissipation\;factor=\;\frac{G^{\prime \prime }}{G\prime }$$

### 2.4. Preparation of Dermatomed Pig Ear Skin

For ex vivo substantivity tests and penetration experiments, we used porcine ear skin. Porcine skin is frequently used as a model for human skin [24,25,26]. Ears of German Land Race pigs with an age between 15 and 30 weeks and a weight of 40–65 kg were used. Pig ears were donated from the Department of Experimental Medicine, University Hospital of Tübingen. The ears were removed right after the death of the animals. Afterwards, the ears were washed with isotonic saline and postauricular skin was removed. The obtained skin sample was cleaned with cotton swabs and isotonic saline. Afterwards the surface was patted dry and packed in aluminium foil. Porcine skin was stored at −30 °C. Before use, the skin was thawed at room temperature. Strips with adequate width were cut and attached onto a styrofoam block. A dermatom (Dermatom GA 630, Aesculap AG and Co. KG, Tuttlingen, Germany) was used to obtain skin samples with a defined thickness of 1 mm. This method is described in previous work of our group [1,2,9,10,27]. For ex vivo substantivity tests pieces with a diameter of 30 mm, and for penetration experiments discs with a diameter of 25 mm were punched out of the skin.

### 2.5. Ex Vivo Substantivity Test

To test the substantivity of the formulations, a method of Herrmann et al. [10] was used. Two heatable steel cylinders were fixed to a texture analyser (Type BDO-FB0.5TS, Zwick Roell GmbH and Co. KG, Ulm, Germany). Both parts were heated to 32 °C (skin surface temperature). The lower part was rigid, while the second cylinder was placed above the other and was movable. A piece of dermatomed pig ear skin was fixed to the lower cylinder and about 4 mg of formulation was applied to the skin surface. To this end, approximately 8 mg of the formulation was put on a plastic cylinder and weighed exactly. By pressing down and rotating, the formulation was transferred to the skin. Afterwards the application aid was weighed again. The mass difference gave the applied mass of formulation. Subsequently, the formulation had 10 min to dry. Another piece of skin or a piece of cloth was fixed to the upper steel cylinder. Then, the two devices were pressed together with a defined force of 10 N for 50 s. The skin surface was cleaned twice with a cotton swab, then soaked with 2 mL of phosphate-buffered saline (PBS) with pH 7.4. The swabs were collected in a 50 mL centrifuge tube and 4 mL of methanol was added to extract the API. Clothing was extracted in a mixture of methanol and PBS in equal parts. Nonivamide was quantified by HPLC. Experiments were performed in triplicate.

### 2.6. In Vivo Substantivity Test

A square of 9 cm^2^ was marked on the upper right arm of female Caucasian volunteers. All subjects (aged 29 ± 6 years) gave their informed consent for inclusion before they participated in the study. The study was conducted in accordance with the Declaration of Helsinki, and the protocol was approved by the local Ethics Committee on Human Research (University of Belgrade, Faculty of Pharmacy, Serbia; approval number: 2298/2, issued on December 2017). Approximately 18 mg of formulation was applied and the exact mass was obtained by difference weighing. After 10 min drying time, a piece of cloth (6 × 6 cm) was fixed in the inner side of the clothing item with safety pins, such that the applied formulation was covered. Afterwards the participants had 3 h at their disposal, to return to their normal physical activities. Then the formulation was washed away twice with a cotton swab soaked with 2 mL of purified water. The two swabs were collected in a centrifuge tube and 10 mL of methanol was added to extract the API. The piece of cloth was put into a second centrifuge tube and 10 mL of methanol and 4 mL of purified water were added. Subsequently, the centrifuge tubes were shaken for 1 min and then put into an ultrasonic bath for 60 min. The amount of formulation was analysed using a UV/Vis spectrometer. All formulations contained 0.9% of avobenzone. This sun protection factor remained on the skin surface and the penetrated amount was negligible [28]. Only volunteers with a recovery within 65–125% of the applied amount of avobenzone were included. The actual determined amount of avobenzone was set as 100%.

### 2.7. Ex Vivo Penetration Experiments

The dermatomed pig ear skin with a thickness of 1 mm was placed on the top of the acceptor compartment of a Franz diffusion cell with 2 cm^2^ penetration area (Gauer Glas, Puettlingen, Germany). The acceptor compartment was filled with a solution of 4% bovine serum albumin in phosphate-buffered saline with pH 7.4. This acceptor medium is equivalent to whole blood with regard to the permeation rate for lipophilic compounds [29]. The diffusion cells were placed in a water bath and heated to 32 °C for 30 min before the formulation was applied. The acceptor medium was stirred at 500 rpm during the incubation time. The experiments were performed under finite dose conditions. To this end, about 4 mg of formulation was applied to the skin surface in the same way as described for substantivity testing (Section 2.5). After 4 h the skin was removed from the diffusion cell. To remove the remaining formulation, skin surface was washed twice with a cotton swab soaked with 2 mL saturated magnesium sulphate solution. The actual penetration area was punched out, directly weighed and frozen in liquid nitrogen. The skin was sectioned horizontally with a cryo-microtome (HM 560 Cryo-Star; Thermo Fisher Scientific, Inc., Waltham, MA, USA) into thin sections with a thickness of 16 µm. The first incomplete cuts and the first complete cut were collected in a 2 mL tube, representing the stratum corneum. The next 14 sections were collected in a second container, representing the viable epidermis. The last fraction, the dermis, was also cut in 16 µm thin sections and put in a third vial. This method has been described in detail by Lunter and Daniels [2]. To extract the API from the skin tissue, 1 mL saturated solution of magnesium sulphate was added. This aqueous phase was covered with 200 µL acetonitrile. The organic liquid contained avobenzone as an internal standard. The tubes were shaken for 60 min at 1000 rpm (BioShake iQ, Analytik Jena AG, Jena, Germany). To achieve complete phase separation, the samples were centrifuged for 15 min at 13,400 rpm (MiniSpin, Eppendorf AG, Hamburg, Germany). Afterwards, 80 µL of the upper organic phase was transferred into a HPLC vial. Nonivamide was quantified by HPLC. This procedure was performed to concentrate nonivamide in the organic phase in order to improve quantification. Experiments were performed in triplicate.

### 2.8. UV/Vis Detection of Avobenzone

The concentration of avobenzone was quantified using a UV/Vis Spectrophotometer Evolution 300 in combination with Visionpro Software (Thermo Scientific, Waltham, MA, USA). The solution was filled in a quartz cuvette. The detection wavelength was 360 nm. If the signal was outside the calibration range, the stock solution was diluted with fresh solvent. The coefficient of determination was >0.999, the limit of detection was 0.009 µg/mL and the limit of quantification 0.033 µg/mL.

### 2.9. HPLC Analytics

The used HPLC system had the following components: Dionex P680 HPLC Pump, Dionex Autosampler ASI-100 Automated Sample Injector, Gynkotek UVD170U Detector (Dionex Corporation, Sunnyvale, CA, USA) and the column-oven SunTherm 5-100 (SunChrom Wissenschaftliche Geräte GmbH, Friedrichsdorf, Germany). For nonivamide quantification in methanol 50% (substantivity measurements), a multistep gradient with acetonitrile and phosphoric acid (pH 3.0) was performed. The amount of acetonitrile was raised from 30 to 75% within 8 min and was then held constant for 2 min. Afterwards the amount was reduced to 30% within 2 min and equilibrated for 3 min. The flow rate was set to 1.15 mL/min, the injection volume was 200 µL and the oven temperature was 40 °C. The retention time of nonivamide was approximately 5.6 min. The HPLC column Nucleosil 100-5 C8 EC 125/4 (Macherey-Nagel GmbH and Co. KG, Dueren, Germany) was used together with the HPLC-precolumn EC 4/3 Universal RP (Macherey-Nagel GmbH and Co. KG, Dueren, Germany). Avobenzone was used as an internal standard. Calibration data are given in Table 2.

To separate and quantify nonivamide and avobenzone in acetonitrile (penetration experiments) the HPLC column Nucleosil 100-5 C18 EC 125/4 (Macherey-Nagel GmbH and Co. KG, Dueren, Germany) was used together with the HPLC-precolumn EC 4/3 Universal RP (Macherey-Nagel GmbH and Co. KG, Dueren, Germany). A multistep gradient with methanol and phosphoric acid (pH 3.0) was performed. At the beginning, the organic amount was set to 45%, reaching 99% after 15 min, and it was then held constant for 2 min. The gradient returned within 2 min to initial conditions and was equilibrated for 5 min. The pump was set to 1.0 mL/min, the injection volume was 60 µL and the oven temperature was 30 °C. The retention time was approximately 8.6 min for nonivamide and approximately 15.1 min for avobenzone. Calibration data are given in Table 2 and Table 3.

### 2.10. Droplet Size Measurements

Droplet size was measured by laser diffraction using Mastersizer^®^ 2000 with Hydro 2000s (Malvern Panalytical Ltd., Malvern, UK). Therefore, 200 µL of the emulsion was added to about 10 mL of purified water and mixed manually. The mixture was put in Hydro 2000s under stirring (2000 rpm) until an obscuration between 10% and 20% was reached. After 60 s the measurement was started. One measurement cycle recorded the signal three times for 30 s. For each emulsion three aliquots were measured. For each aliquot three repetitions were performed.

### 2.11. Statistical Analysis

The data were obtained from repeated measurements (*n* ≥ 3). The diagrams show mean ± standard deviation (SD). The data were analysed by unpaired *t*-test (*p* < 0.05) or, if multiple groups were to be compared, by one-sided one factorial analysis of variance (ANOVA) (*p* < 0.05) followed by the Student–Newman–Keuls test.

## 3. Results and Discussion

**Figure 1:**
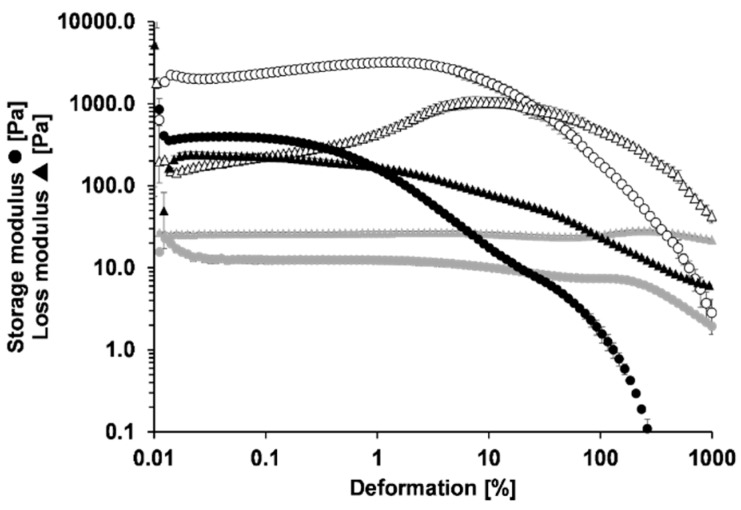
Oscillatory measurements of HNC at 32 °C (black icons) and the emulsion containing 4.8% methyl cellulose at 5 °C (grey icons) and 32 °C (open icons), mean ± SD, *n* = 3.

The aim of this work was to develop methyl cellulose-stabilised emulsions that show enhanced substantivity due to thermogelation. We thus investigated the effect of different methyl cellulose concentrations and different macrogol types on rheological properties and substantivity. The semi-solid Hydrophilic Nonivamide Cream (HNC) that is used as a therapy for chronic itch was used for comparison. To determine loss and storage modulus, oscillatory experiments were performed. The results gave information on the viscoelastic properties of the formulation. Figure 1 exemplarily shows the results of the oscillatory experiments at 5 and at 32 °C, which are equivalent to storage temperature and skin surface temperature, respectively. At 5 °C the presented emulsion has a higher loss modulus compared to its storage modulus and thus the formulation is a liquid preparation at this temperature. By increasing the temperature to 32 °C, the rheological parameters change. Storage and loss modulus reach higher values. Here, the storage modulus is higher than the loss modulus. This means that the preparation has semi-solid properties at skin surface temperature. Therefore, it can be concluded that the developed formulations show thermogelling properties. The emulsion has a dissipation factor of about 0.1 and thus, compared to HNC, it is approximately six times smaller. Therefore, the elastic properties of the emulsion are more pronounced compared to those of HNC.

In Table 4, the rheological parameters of different emulsions are listed (for more detailed information the reader is kindly referred to supplementary material Figures S1–S5). With increasing methyl cellulose concentration, the storage and loss modulus increase, but no effect on the dissipation factor appears. The emulsions have a dissipation factor of about 0.1. Apart from different methyl cellulose concentrations, we also investigated the impact of short-chain versus long-chain macrogols. The emulsions with an amount of 0.5% or 1.0% of methyl cellulose but different types of macrogol did not differ in their rheological properties. The emulsion with 4.8% methyl cellulose and macrogol 200 had a higher storage and loss modulus than the emulsion containing macrogol 4000. The latter finding is somewhat contrary to our expectation that the long-chain macrogol that is solid at skin surface temperature might also lead to a more solid gel character. Presumably the short-chain macrogol interacts more strongly with methyl cellulose and thus exhibits a stronger impact on the rheological properties of the emulsion.

The droplet size of the emulsions was determined directly after preparation. The results are shown in Table 5. The emulsions with high methyl cellulose concentrations showed the smallest droplet size. Upon decreasing the polymer concentration, the values for d10, d50 and d90 rose. Methyl cellulose is the emulsifying agent and a direct correlation between droplet size and methyl cellulose concentration was expected. Our results do not show any effect of the macrogol chain length on the droplet size. The emulsions with the same amount of methyl cellulose but different macrogol types were comparable with respect to their droplet size. The macroscopic and microscopic appearances of the emulsions are shown in supplementary Figure S6.

**Figure 2:**
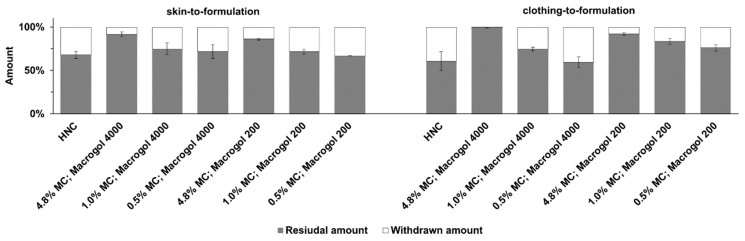
Ex vivo substantivity tests simulating skin-to-formulation and clothing-to-formulation contact. Results of HNC and developed emulsions, mean ± SD, *n* = 3.

Furthermore, the effect of different methyl cellulose concentrations and macrogols on substantivity was investigated. The results of the ex vivo substantivity test are shown in Figure 2. It can be seen that skin-to-formulation or clothing-to-formulation contact removes about one third of HNC. Formulations with 0.5% or 1.0% methyl cellulose showed comparable results. About 70% of the applied emulsion remained at skin surface after simulated contact. The two emulsions with a methyl cellulose concentration of 4.8% showed residual amounts of 86% and 100%. This shows that neither skin-to-formulation nor clothing-to-formulation removed a substantial share of the applied emulsion.

The results of the ex vivo substantivity tests show that with a methyl cellulose concentration of 4.8% and macrogol 4000 or 200, high residual fractions were obtained—i.e., the withdrawn amount was not more than 14% when skin-to-formulation contact was simulated. Moreover, a residual amount up to 100% was obtained, when clothing-to-formulation was simulated. A lower methyl cellulose concentration resulted in higher withdrawn amounts. Emulsions with the same amount of methyl cellulose but different types of macrogol were comparable. The emulsions with a high methyl cellulose concentration were neither affected by simulating skin-to-formulation nor clothing-to-formulation contact. Statistical results of the ex vivo substantivity test are given in the supplementary material (Tables S1 and S2). These results can be explained by the rheological behaviour of the formulations. Emulsions that showed similar rheological behaviour also showed similar substantivity. Emulsions with high methyl cellulose concentration were characterised by their high storage modulus and a low dissipation factor and thus showed high substantivity. On the other hand, emulsions with low methyl cellulose concentration showed low storage modulus and a higher dissipation factor, resulting in lower substantivity. The substantivity of the developed emulsions is comparable to that of previously developed film-forming formulations [9,10]. This finding is remarkable, as the emulsions do not form a deductible, cohesive film.

The droplet size plays a minor role in resistance against contact with other surfaces. Formulations with 4.8%, 1.0% and 0.5% methyl cellulose showed differences in their droplet size, though we could not find a clear trend in their substantivity.

The next step was to investigate the skin penetration of nonivamide from the developed emulsions. The nonivamide site of action is located in the viable epidermis. Thus, ex vivo permeation experiments were not informative in this case as they reflect transdermal absorption. We thus focused on ex-vivo penetration experiments that enabled the measurement of the nonivamide concentration in the different strata of the skin. Penetration from the emulsion was compared to penetration from HNC, which contained nonivamide in a concentration that is clinically used (0.05%). The aim was to achieve drug levels at the site of action that were comparable to this reference. The emulsion contained 0.9% nonivamide, as this concentration had been found to give similar drug concentrations in skin to HNC 0.05% in earlier work by our group [2,3,27].

**Figure 3:**
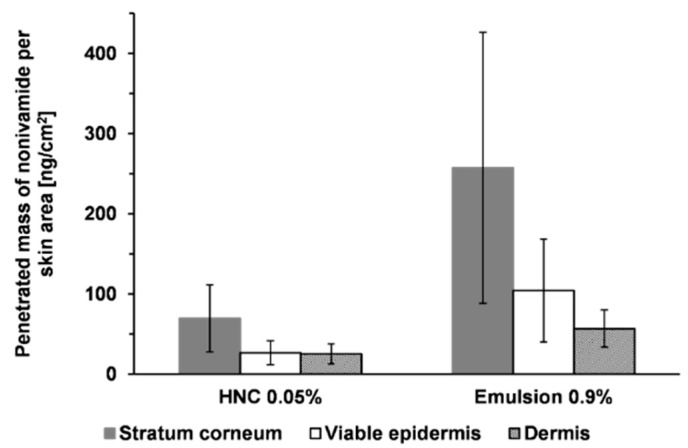
Penetrated amount of nonivamide after 4 h from HNC 0.05% and a developed emulsion containing 0.9% nonivamide, mean ± SD, *n* ≥ 3.

**Figure 4:**
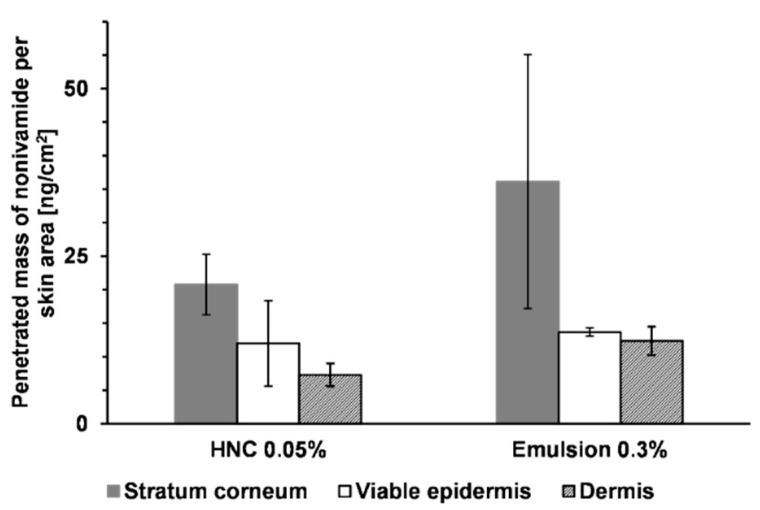
Penetrated amount of nonivamide after 4 h from HNC 0.05% and a developed emulsion containing 0.3% nonivamide, mean ± SD, *n* = 3.

Figure 3 shows the penetrated amount of nonivamide from HNC 0.05% and an emulsion with 0.9% in the different skin layers. From HNC 0.05%, about 70 ng/cm^2^ penetrated in the stratum corneum after 4 h incubation time. In both the viable epidermis and dermis we could find about 25 ng/cm^2^. The new emulsion with an API concentration of 0.9% resulted in a mean of 250 ng/cm^2^ nonivamide in the stratum corneum, 100 ng/cm^2^ in the viable epidermis and 60 ng/cm^2^ in the dermis. The penetrated amount in the stratum corneum and viable epidermis was about three to four times higher compared to that achieved using HNC 0.05%. Therefore, the drug concentration was set to 0.3% in a subsequent experiment. A reduced API concentration of 0.3% led to a reduced penetrated amount (Figure 4). Drug concentration in the viable epidermis, the site of action, was about 14 ng/cm^2^ from the emulsion and 12 ng/cm^2^ from HNC, and was thus comparable to HNC 0.05%. Although the emulsion contained a higher concentration of API, the penetrated amount was almost the same. This can be explained by the composition of the two formulations. HNC contained 15% of propylene glycol and 9% of ethanol, which are both known as penetration enhancers [30,31,32], whereas the emulsion did not contain propylene glycol. Differences in penetrated amount from HNC 0.05% between the two experiments were related to the use of skin from different donors. It can thus be concluded that the emulsion delivers an amount of the active ingredient to the skin that is suitable for therapy. The penetrated amount can be tuned by adjusting the drug concentration in the emulsion.

**Figure 5:**
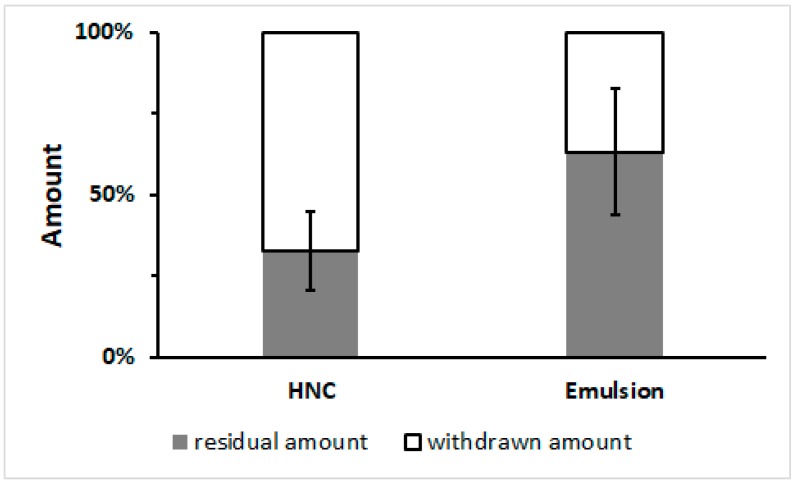
In vivo substantivity tests simulating clothing-to-formulation contact. Results of HNC and the developed emulsion, mean ± SD, *n* = 5 or 6.

Finally, the in vivo substantivity of the thermogelling emulsion was compared to that of HNC. The results are given in Figure 5. It can clearly be seen that from HNC, 67% of the formulation was transferred to the clothing, whereas from the emulsion only 37% was removed. This is in accordance with the ex vivo substantivity test, which had already shown that the emulsion was superior to HNC. Nevertheless, the SDs were higher and the differences between the two formulations were slightly less evident in the in vivo experiment. We believe that this was to be expected as a higher degree of variability is typical for in vivo experiments. As our study group was very homogeneous regarding age, gender and ethnicity, we believe that the variability manly reflects different physical activity among the volunteers, who were not given any restriction on to how to behave during the application interval. Therefore, the in vivo experiment better reflects real life conditions compared to the ex vivo experiment. As we could still find a significant difference between substantivity in the in vivo experiment, we are convinced that the emulsion will be superior to HNC under real-life conditions.

## 4. Conclusions

Thermogels or formulations that form a gel in situ are promising dosage forms for different applications. Ophthalmic, injectable as well as dermal preparations are described in the literature. Lin et al. investigated thermogels containing mixtures of alginate and pluronics for ophthalmic application [33]. Zhang et al. used PLGA-PEG_PLGA-block-copolymers as injectable and biodegradable delivery systems [34]. In the context of dermal application, Hemelrijk et al. employed poloxamer to form thermogels for the enhanced delivery of 5-ALA into keratotic skin [35]. Takeuchi et al. investigated methyl cellulose-based thermogels and characterised the impact of different excipients on rheological parameters [22]. Their gels contained no oil phase and no studies concerning the dermal uptake of pharmaceutical actives from the gels were conducted. Further, the question of substantivity has not been investigated in publications dealing with methyl cellulose thermogels. Although researchers and practitioners are aware of the fact that a substantial amount of formulations is removed from the skin after application, research on the rational development of formulations with enhanced substantivity is scarce.

The presented work illustrates that the developed thermogelling emulsions are more substantive than the tested conventional semi-solid cream. With a high methyl cellulose concentration, almost the entire applied formulation stays at the application site. The results suggest that a connection between rheological characteristics and substantivity exists. High storage modulus and low dissipation factor increase the residual amount of topical formulations. Thus, it is possible to combine the advantages of the high substantivity of film-forming formulations and the beneficial characteristics of conventional semi-solid creams with regard to skin care. While in film-forming formulations the oil is immobilised in mesoporous silica particle [1], in the presented emulsions the medium-chain triglycerides are available for skin care. The penetrated amount of nonivamide is comparable to the monographed HNC 0.05%. The thermogelling emulsions may therefore provide an alternative to conventional creams in the therapy of chronic skin diseases. With enhanced substantivity, they may reduce application frequency and the contamination of patients’ environment with the active ingredients. They may also be used as a formulation platform for other pharmaceutical actives. Future research will focus on the impact of further excipients on thermogelation and on the selection of optimal additives for the enhancement of in vivo substantivity. As differences were found between ex vivo and in vivo substantivity, we will further improve the ex vivo method of substantivity determination to better reflect the in vivo situation. Furthermore, the optimisation of drug delivery properties will be investigated.

## Figures and Tables

**Table 1:** Percentage composition of thermogelling emulsions. The amount of nonivamide varied between 0.3 and 0.9% with regard to the final mass of the formulation.

	A	B	C
Substance	Amount (% m/m)		
Methyl cellulose	0.5	1.0	4.8
Sodium citrate	2.1	2.1	2.1
Macrogol 200 or 4000	6.0	6.0	6.0
Ethanol	9.0	9.0	9.0
Medium-chain triglycerides with nonivamide	25.0	25.0	25.0
Purified water	to 100	to 100	to 100

The composition and preparation of Hydrophilic Nonivamide Cream (HNC) is described in “Neues Rezeptur-Formularium”. The cream was prepared as described in the monograph [23]. Capsaicin was replaced by nonivamide.

**Table 2:** Calibration data for nonivamide in methanol 50% and acetonitrile; *n* = 3.

Solvent	Calibration Range (µg/mL)	Coefficient of Determination (*R*^2^)	Limit of Detection (µg/mL)	Limit of Quantification (µg/mL)
Methanol 50%	0.01–0.10	0.9868	0.014	0.042
Methanol 50%	0.1–1.0	0.9953	0.029	0.103
Methanol 50%	1.0–10.0	0.9949	0.304	1.066
Acetonitrile	0.05–0.50	0.9878	0.023	0.079
Acetonitrile	0.1–1.0	0.9911	0.042	0.146
Acetonitrile	1.0–10.0	0.9994	0.109	0.390

**Table 3:** Calibration data for avobenzone in acetonitrile; *n* = 3.

Solvent	Calibration Range (µg/mL)	Coefficient of Determination (*R*^2^)	Limit of Detection (µg/mL)	Limit of Quantification (µg/mL)
Acetonitrile	0.05–0.50	0.9971	0.011	0.040
Acetonitrile	0.1–1.0	0.9954	0.031	0.109
Acetonitrile	1.0–10.0	0.9975	0.230	0.816

**Table 4:** Rheological parameters of thermogelling emulsions and HNC at 32 °C, mean ± SD, *n* ≥ 3.

Formulation	Storage Modulus (Pa)	Loss Modulus (Pa)	Dissipation Factor
HNC	396.73 (±32.32)	233.37 (±16.95)	0.59 (±0.01)
4.8% Methyl cellulose, macrogol 4000	284.00 (±147.55)	25.43 (±8.96)	0.09 (±0.01)
1.0% Methyl cellulose, macrogol 4000	5.64 (±1.11)	0.61 (±0.27)	0.10 (±0.04)
0.5% Methyl cellulose, macrogol 4000	4.28 (±2.07)	0.44 (±0.23)	0.14 (±0.12)
4.8% Methyl cellulose, macrogol 200	2123.33 (±51.32)	200.00 (±6.24)	0.09 (±0.00)
1.0% Methyl cellulose, macrogol 200	3.52 (±0.62)	0.42 (±0.08)	0.12 (±0.04)
0.5% Methyl cellulose, macrogol 200	2.00 (±0.31)	0.44 (±0.23)	0.23 (±0.12)

**Table 5:** Droplet size of the emulsions with different methyl cellulose concentrations and different macrogol types. Formulations were prepared without nonivamide, mean ± SD, *n* = 3.

Formulation	d10	d50	d90
4.8% Methyl cellulose, macrogol 4000	1.34 (±0.01)	2.37 (±0.00)	3.82 (±0.01)
1.0% Methyl cellulose, macrogol 4000	1.93 (±0.01)	4.88 (±0.01)	9.33 (±0.03)
0.5% Methyl cellulose, macrogol 4000	3.32 (±0.04)	6.70 (±0.07)	12.37 (±0.16)
4.8% Methyl cellulose, macrogol 200	1.40 (±0.00)	2.26 (±0.01)	3.45 (±0.02)
1.0% Methyl cellulose, macrogol 200	2.74 (±0.06)	5.95 (±0.08)	11.14 (±0.21)
0.5% Methyl cellulose, macrogol 200	3.09 (±0.11)	6.49 (±0.11)	11.99 (±0.21)

## Supplementary Materials

The following are available online at https://www.mdpi.com/1999-4923/11/8/361/s1, Figure S1: Oscillatory measurements methyl cellulose stabilised emulsion at 5 °C (grey icons) and 32 °C (open icons). The emulsion contains Macrogol 4000, quantities corresponds to emulsion A in Table 1, mean ± SD, *n* = 3; Figure S2: Oscillatory measurements methyl cellulose stabilised emulsion at 5°C (grey icons) and 32 °C (open icons). The emulsion contains Macrogol 4000, quantities corresponds to emulsion B in Table 1, mean ± SD, *n* = 3; Figure S3: Oscillatory measurements methyl cellulose stabilised emulsion at 5°C (grey icons) and 32 °C (open icons). The emulsion contains Macrogol 4000, quantities corresponds to emulsion C in Table 1, mean ± SD, *n* = 3; Figure S4: Oscillatory measurements methyl cellulose stabilised emulsion at 5°C (grey icons) and 32 °C (open icons). The emulsion contains Macrogol 200, quantities corresponds to emulsion A in Table 1, mean ± SD, *n* = 3; Figure S5: Oscillatory measurements methyl cellulose stabilised emulsion at 5°C (grey icons) and 32 °C (open icons). The emulsion contains Macrogol 200, quantities corresponds to emulsion B in Table 1, mean ± SD, *n* = 3; Figure S6: left: macroscopic image of a thermogelling emulsion; right: microscopic image of a thermogelling emulsion; Table S1: Statistically significance for residual amount simulating skin-to formulation contact. Emulsions are named by their methyl cellulose concentration and the molecular weight of the used macrogol. The data were analysed by one-sided one factorial analysis of variance (ANOVA) (*p* < 0.05) followed by Student-Newman-Keuls test. Lines which are not linked with the same letter are significant different; Table S2: Statistically significance for residual amount simulating clothing-to formulation contact. Emulsions are named by their methyl cellulose concentration and the molecular weight of the used macrogol. The data were analysed by one-sided one factorial analysis of variance (ANOVA) (*p* < 0.05) followed by Student-Newman-Keuls test. Lines which are not linked with the same letter are significant different.
