# Supplementary material for: Optimization of Rheological Behaviour and Skin Penetration of Thermogelling Emulsions with Enhanced Substantivity for Potential Application in Treatment of Chronic Skin Diseases
Source: Pharmaceutics. 2019 Jul 24;11(8):361. doi: 10.3390/pharmaceutics11080361 (PMC6723268; doi:10.3390/pharmaceutics11080361)
Supplement: Supplementary file 1 [file pharmaceutics-11-00361-s001.pdf]

pharmaceutics

MDPI

Supplementary Materials: Optimization of Rheological Behaviour and Skin Penetration of Thermogelling Emulsions with Enhanced Substantivity for Potential Application in Treatment of Chronic Skin Diseases

Markus Schmidberger [1], Ines Nikolic [2], Ivana Pantelic [2] and Dominique Jasmin Lunter [1,*]

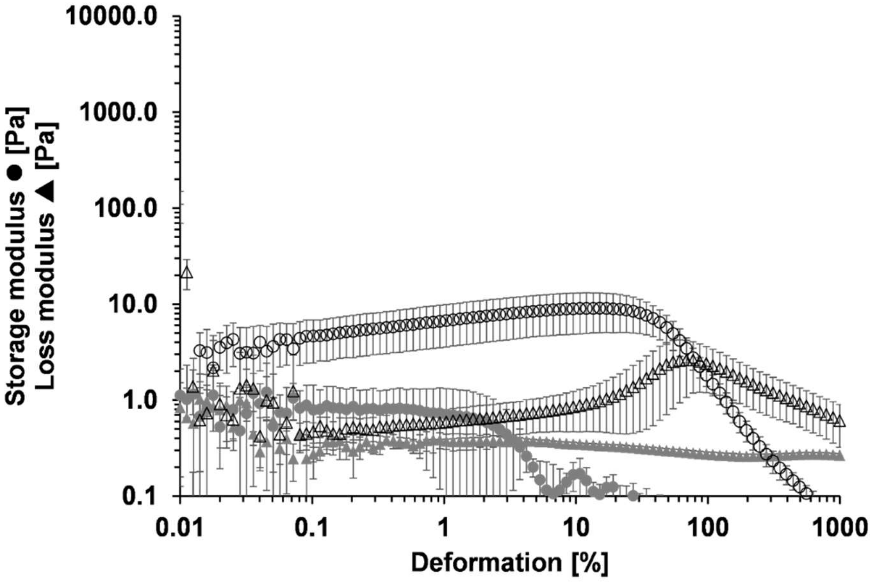

Figure S1. Oscillatory measurements methyl cellulose stabilised emulsion at 5°C (grey icons) and 32 °C (open icons). The emulsion contains Macrogol 4000, quantities corresponds to emulsion A in Table 1, mean ± SD, *n* = 3.

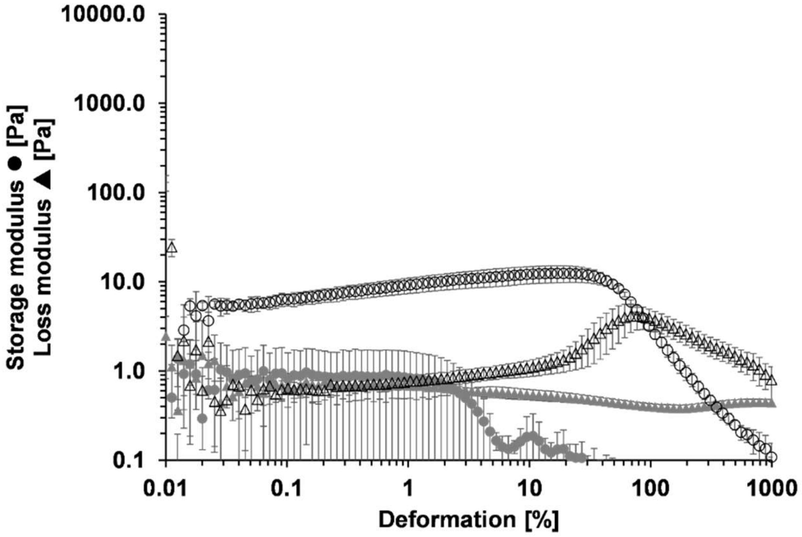

Figure S2. Oscillatory measurements methyl cellulose stabilised emulsion at 5°C (grey icons) and 32 °C (open icons). The emulsion contains Macrogol 4000, quantities corresponds to emulsion B in Table 1, mean ± SD, *n* = 3.

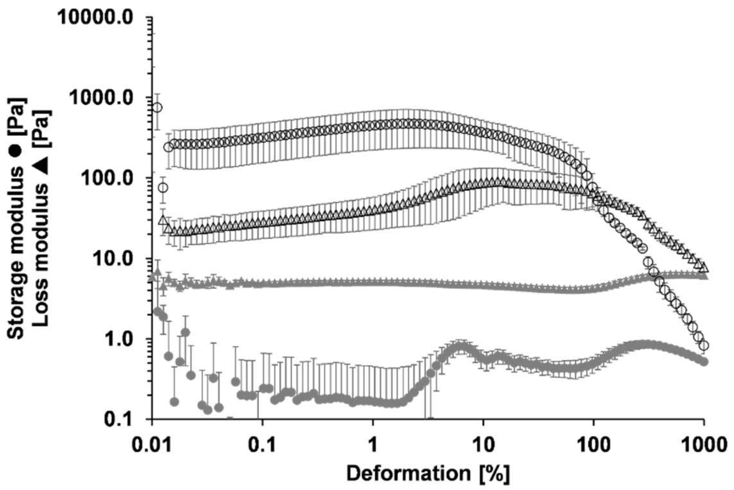

Figure S3. Oscillatory measurements methyl cellulose stabilised emulsion at 5°C (grey icons) and 32 °C (open icons). The emulsion contains Macrogol 4000, quantities corresponds to emulsion C in Table 1, mean ± SD, *n* = 3.

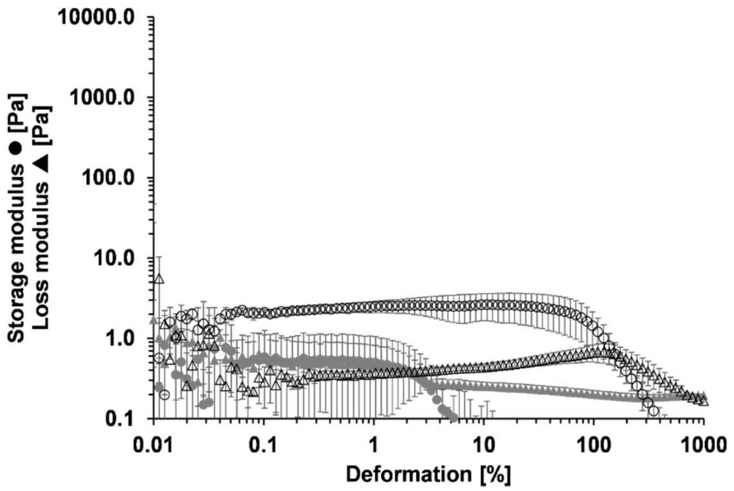

Figure S4. Oscillatory measurements methyl cellulose stabilised emulsion at 5°C (grey icons) and 32 °C (open icons). The emulsion contains Macrogol 200, quantities corresponds to emulsion A in Table 1, mean ± SD, *n* = 3.

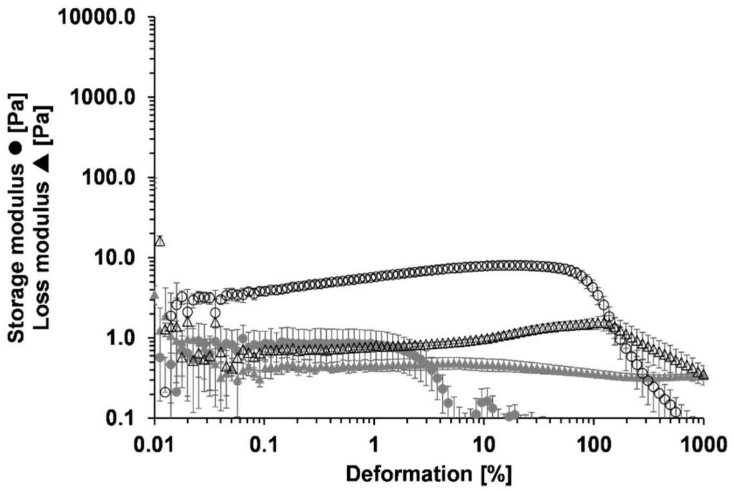

Figure S5. Oscillatory measurements methyl cellulose stabilised emulsion at 5°C (grey icons) and 32 °C (open icons). The emulsion contains Macrogol 200, quantities corresponds to emulsion B in Table 1, mean ± SD, *n* = 3.

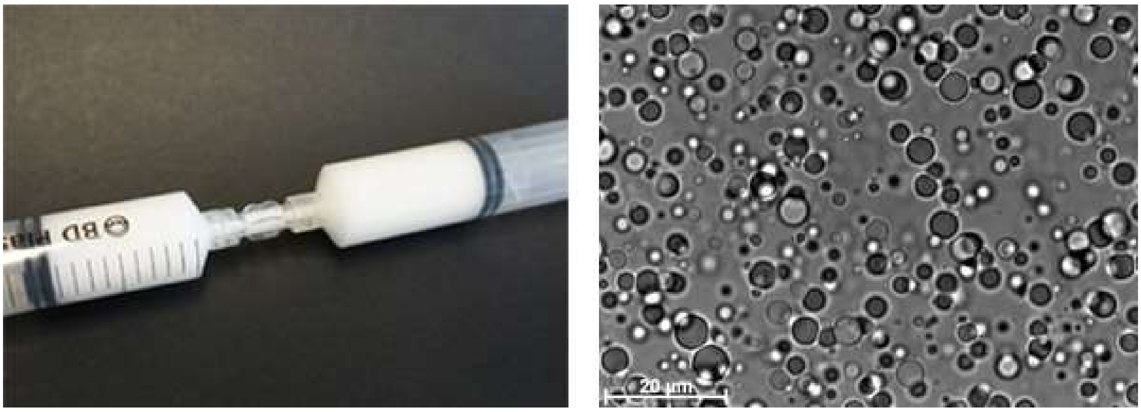

Figure S6. left: macroscopic image of a thermogelling emulsion; right: microscopic image of a thermogelling emulsion.

Table S1. Statistically significance for residual amount simulating skin-to formulation contact. Emulsions are named by their methyl cellulose concentration and the molecular weight of the used macrogol. The data were analysed by one-sided one factorial analysis of variance (ANOVA) ($p < 0.05$) followed by Student-Newman-Keuls test. Lines which are not linked with the same letter are significant different.

Formulation			Mean [%]
4.8% MC, Macrogol 4000	A		91.53
4.8% MC, Macrogol 200	A		86.13
1.0% MC, Macrogol 4000		B	75.00
1.0% MC, Macrogol 200		B	71.92
0.5% MC, Macrogol 4000		B	71.74
HNC		B	68.00
0.5% MC, Macrogol 200		B	66.69

Table S2. Statistically significance for residual amount simulating clothing-to formulation contact. Emulsions are named by their methyl cellulose concentration and the molecular weight of the used macrogol. The data were analysed by one-sided one factorial analysis of variance (ANOVA) ($p < 0.05$) followed by Student-Newman-Keuls test. Lines which are not linked with the same letter are significant different.

Formulation					Mean [%]
4.8% MC, Macrogol 4000	A				99.89
4.8% MC, Macrogol 200	A	B			92.16
0.5% MC, Macrogol 200		B	C		84.27
0.5% MC, Macrogol 200			C		76.19
1.0% MC, Macrogol 4000			C		74.55
HNC				D	60.51
0.5% MC, Macrogol 4000				D	59.65
